# TBC1D12 is a novel Rab11-binding protein that modulates neurite outgrowth of PC12 cells

**DOI:** 10.1371/journal.pone.0174883

**Published:** 2017-04-06

**Authors:** Mai E. Oguchi, Kenta Noguchi, Mitsunori Fukuda

**Affiliations:** Laboratory of Membrane Trafficking Mechanisms, Department of Developmental Biology and Neurosciences, Graduate School of Life Sciences, Tohoku University, Aobayama, Aoba-ku, Sendai, Miyagi, Japan; Institut Curie, FRANCE

## Abstract

Recycling endosomes are generally thought to play a central role in endocytic recycling, but recent evidence has indicated that they also participate in other cellular events, including cytokinesis, autophagy, and neurite outgrowth. Rab small GTPases are key regulators in membrane trafficking, and although several Rab isoforms, e.g., Rab11, have been shown to regulate recycling endosomal trafficking, the precise mechanism by which these Rabs regulate recycling endosomes is not fully understood. In this study, we focused on a Rab-GTPase-activating protein (Rab-GAP), one of the key regulators of Rabs, and comprehensively screened 43 mammalian Tre-2/Bub2/Cdc16 (TBC)/Rab-GAP-domain-containing proteins (TBC proteins) for proteins that specifically localize on recycling endosomes in mouse embryonic fibroblasts (MEFs). Four of the 43 mammalian TBC proteins screened, i.e., TBC1D11, TBC1D12, TBC1D14, and EVI5, were found to colocalize well with transferrin receptor, a well-known recycling endosome marker. We further investigated the biochemical properties of TBC1D12, a previously uncharacterized TBC protein. The results showed that TBC1D12 interacted with active Rab11 through its middle region and that it did not display Rab11-GAP activity *in vitro*. The recycling endosomal localization of TBC1D12 was found to depend on the expression of Rab11. We also found that TBC1D12 expression had no effect on common Rab11-dependent cellular events, e.g., transferrin recycling, in MEFs and that it promoted neurite outgrowth, a specialized Rab11-dependent cellular event, of PC12 cells independently of its GAP activity. These findings indicated that TBC1D12 is a novel Rab11-binding protein that modulates neurite outgrowth of PC12 cells.

## Introduction

Cells respond to changes in the extracellular environment by internalizing extracellular materials, lipids, and cell surface proteins by endocytosis, and the internalized substances are transported to lysosomes for degradation or recycled back to the plasma membrane for reuse (so-called endocytic recycling) [1, 2]. Recycling endosomes are generally thought to play a pivotal role in endocytic recycling [2], but recent studies have indicated that recycling endosomes also regulate a variety of cellular events, including cytokinesis [3, 4], autophagy [5], and neurite outgrowth [6–11], although the precise mechanisms by which they regulate these events are not fully understood.

Rab small GTPases are key regulators of membrane trafficking in recycling endosomes [12]. Rabs belong to the Ras-like superfamily and constitute the largest family of membrane trafficking proteins in eukaryotes [13–15]. Mammals contain approximately 60 different Rab isoforms, and each isoform is thought to regulate a specific type (or step) of membrane trafficking. Like other small GTPases, Rabs function as molecular switches by cycling between two different guanine-nucleotide-binding states, a GTP-bound active state and a GDP-bound inactive state. The cycle is thought to be strictly controlled by two regulatory enzymes, a guanine nucleotide exchange factor (GEF) [16, 17] and a GTPase-activating protein (GAP) [16, 18]. In their active state, Rabs localize on the surface of vesicles or organelles and regulate their trafficking by recruiting their specific effector molecule(s) [13–15]. Thus, the identification and characterization of Rab-GEFs and Rab-GAPs is an important step toward understanding the spatiotemporal regulation of Rab small GTPases, and yet analysis of their functions in the past has been very limited.

In contrast to the presence of multiple types of Rab-GEFs (i.e., each type of GEF contain a different GEF domain) [17], all of the Rab-GAPs identified thus far except Rab3-GAP have contained a Tre-2/Bub2/Cdc16 (TBC) domain, which is thought to activate the intrinsic GTPase activity of Rabs [18]. More than 40 different TBC-domain-containing proteins (referred to as TBC proteins hereafter) have been identified in humans and mice [18], but because of their large numbers their localization and functions remain to be determined. Actually, no attempt has ever been made to systematically screen for Rab-GAPs that localize and function on recycling endosomes.

In this study, we comprehensively screened 43 mammalian TBC proteins for proteins that colocalize with transferrin receptor (TfR), a well-known recycling endosome marker, and we identified TBC1D12 as a novel recycling endosome-resident protein. The results of biochemical and deletion analyses indicated that TBC1D12 interacts with Rab11 through its middle region rather than its C-terminal TBC domain and that it is recruited to recycling endosomes in a Rab11-dependent manner. We also investigated possible involvement of TBC1D12 in several Rab11-dependent cellular events, including transferrin (Tf) recycling and neurite outgrowth, and discovered that TBC1D12 is a novel modulator of neurite outgrowth of PC12 cells. We discuss the possible functions of TBC1D12 in recycling endosomal trafficking based on our findings.

## Materials and methods

### Antibodies and siRNAs (small interfering RNAs)

Anti-Rab11 mouse monoclonal antibody (#610657, BD Transduction Laboratories, Lexington, KY), anti-TfR mouse monoclonal antibody (#13–6800, ZYMED Laboratories Inc., San Francisco, CA), anti-β-actin mouse monoclonal antibody (#G043, Applied Biological Materials Inc., Richmond, BC, Canada), anti-green fluorescent protein (GFP) rabbit polyclonal antibody (#598, MBL, Nagoya, Japan), anti-TBC1D12 rabbit antibody (#HPA038277, Sigma-Aldrich, St. Louis, MO), horseradish peroxidase (HRP)-conjugated anti-FLAG tag (M2) mouse monoclonal antibody (#A8592, Sigma-Aldrich), HRP-conjugated anti-T7 tag mouse monoclonal antibody (#69048-3CN, Merck KGaA, Darmstadt, Germany), HRP-conjugated anti-GFP rabbit polyclonal antibody (#598–7, MBL), and Alexa Fluor 488/594-conjugated secondary antibodies (Thermo Fisher Scientific Corp., Hudson, NH) were obtained commercially. Anti-LC3β (simply referred to as LC3 throughout this paper) rabbit polyclonal antibody was affinity-purified as described previously [19]. Rab11A/B siRNAs were prepared as described previously [11]. Stealth RNAi^™^ siRNAs (simply referred to as siRNAs hereafter) against rat TBC1D12 (target site: 5’-AAUACUUCAACAUCAUGCUGUGAUC-3’ [siTBC1D12 #1] and 5’-AUUAAUUCCAGACUAGCCUCUCGGU [siTBC1D12 #2]) were purchased from Life Technologies.

### cDNA cloning and plasmid construction

cDNAs encoding each of the human or mouse TBC proteins except TBC1D28 and TBC1D29 were cloned as described previously [20–22]. The cDNAs encoding human TBC1D28 and TBC1D29 were obtained from Marathon-Ready human brain and/or testis cDNA (Clontech-Takara Bio Inc., Shiga, Japan) by performing PCR with the following pairs of oligonucleotides as described previously [23]: 5’-GGATCCATGGAGATGGATGAGGACCC-3’ (TBC1D28 Met primer; *Bam*HI linker underlined) and 5’-**TCA**AGAAGTTGCAACACCCC-3’ (TBC1D28 stop primer; stop codon in bold), and 5’-GGATCCATGGGGCATCTGGACAAGGA-3’ (TBC1D29 Met primer; *Bam*HI linker underlined) and 5’-**TTA**CCCTATTATCCTGCCAC-3’ (TBC1D29 stop primer; stop codon in bold), respectively. The cDNA of each TBC protein was subcloned into the *Bgl*II/*Sal*I site or *Bgl*II/*Eco*RI site of the pEGFP-C1 vector (Clontech-Takara Bio Inc.). Three truncation mutants of TBC1D12, i.e., TBC1D12-N (amino acids [AA] 1–277), TBC1D12-M (AA 278–490), and TBC1D12-TBC (AA 470–781), were produced by performing PCR using the following pairs of oligonucleotides (*Bam*HI linker underlined and stop codons in bold): 5’-GGATCCATGGTGGGTCCGGAGGATG-3’ and 5’-**CTA**GAAGTGAATGTCAGAAAAGCCCA-3’ for TBC1D12-N; 5’-GGATCCAACTCTCGCAACACGTTCCA-3’ and 5’-**CTA**TCCCTGCCACCACAATTCTCGAA-3’ for TBC1D12-M; 5’-GGATCCATACTGCCCAATTGGGAAGT-3’ and 5’-**CTA**GCTTTTCAAAGCAGGA-3’ for TBC1D12-TBC. A GTPase-deficient TBC1D12 mutant, TBC1D12 (R559K), was produced by conventional two-step PCR techniques as described previously [24]. The resulting TBC1D12 truncation mutants and point mutant were subcloned into the pEF-T7 vector (modified from pEF-BOS) [23] and/or the pEGFP-C1 vector. A constitutively active form and a constitutively negative form of Rab11A, Rab11A-Q70L and Rab11A-S25N, respectively, were prepared as described previously [20, 25] and subcloned into the pmStr (monomeric Strawberry)-C1 vector [26] and the pGEX-4T-3 vector (GE Healthcare, Little Chalfont, Buckinghamshire, UK). The plasmids used in this study are available from RIKEN BioResource Center (http://dna.brc.riken.jp/DataSheet/DEP005893).

### Cell cultures and transfections

Mouse embryonic fibroblasts (MEFs) were a generous gift from Dr. Noboru Mizushima (The University of Tokyo, Tokyo, Japan). Plat-E cells were donated by Dr. Toshio Kitamura (The University of Tokyo). MEFs and COS-7 cells were cultured at 37°C in Dulbecco’s modified Eagle’s medium (DMEM) (Wako Pure Chemical Industries, Ltd., Osaka, Japan) supplemented with 10% fetal bovine serum (FBS), 100 units/mL penicillin G, and 100 μg/mL streptomycin under 5% CO_2_. One day after plating, plasmids and siRNAs were transfected into MEFs by using Lipofectamine 2000 and RNAiMAX (Thermo Fisher Scientific Corp.), respectively, each according to its manufacturer’s instructions. Transfection of plasmids into COS-7 cells was similarly achieved by using Lipofectamine LTX/Plus (Thermo Fisher Scientific Corp.) according to the manufacturer’s instructions. PC12 cell cultures and transfections were performed as described previously [27]. Plat-E cell cultures and retrovirus infection were performed as described previously [28].

### Immunofluorescence and image analyses

Cells that had been transfected with plasmids and/or siRNAs were fixed with 4% paraformaldehyde (PFA) for 15–20 min at room temperature and permeabilized with 0.3% Triton X-100. Immunostaining was performed with the specific primary antibodies and Alexa Fluor 488/594-conjugated secondary antibodies. The immunostained cells were examined with a confocal fluorescence microscope (FV1000 or FV1000-D; Olympus, Tokyo, Japan) or with an all-in-one fluorescence microscope (BZ-X700; Keyence, Osaka, Japan).

For the transferrin (Tf) uptake assays, after pre-culturing transfected MEFs in unsupplemented DMEM for 2 h at 37°C, the cells were washed twice with ice-cold phosphate-buffered saline (PBS) and cultured in DMEM containing 5 μg/mL Alexa Fluor 594-conjugated Tf (Alexa594-Tf; Thermo Fisher Scientific Corp.) for 1 h on ice. The cells were then washed twice with ice-cold PBS to remove unbound Tf and incubated at 37°C in DMEM containing 10% FBS. After incubation for the times indicated (S3 Fig), the cells were fixed and examined with the confocal fluorescence microscopes described above. The intensity of Alexa594-Tf staining in individual cells was measured with ImageJ software (version 1.49v National Institutes of Health).

To count the numbers of LC3 dots, MEFs cultured on 12-mm coverslips were first transfected with the plasmids indicated. Two days after transfection, the cells were cultured in EBSS (Sigma-Aldrich) for 2 h (“starved” conditions). After fixation, the cells were stained with anti-LC3 antibody, and images were acquired at random with a confocal fluorescence microscope. The number of LC3 dots per cell was counted with the Analyze Particles plugin in ImageJ software by setting “size (pixel2)” = from 4 to infinity and “circularity” = from 0.5 to 1.0.

### Immunoblot analysis

Cells were lysed in a lysis buffer (50 mM HEPES-KOH, pH7.2, 150 mM NaCl, 1 mM MgCl_2_, 1% Triton X-100, and a protease inhibitor cocktail [Roche, Basal, Switzerland]). Total cell lysates were subjected to 8%, 10%, or 12.5% SDS-polyacrylamide gel electrophoresis (PAGE), and proteins were transferred to PVDF membranes (Merck Millipore, Darmstadt, Germany) by electroblotting for 90 min at a constant voltage setting of 50 V. The blots were blocked with 1% skim milk and 0.1% Tween-20 in PBS and then reacted with specific primary antibodies. The immunoreactive bands were visualized with appropriate HRP-conjugated secondary antibodies (Sigma-Aldrich) and detected by enhanced chemiluminescence (ECL).

### GST (glutathione *S*-transferase) pull-down assays

GST-Rab11A-Q70L and GST-Rab11A-S25N were expressed in *Escherichia coli* JM109 and purified by the standard protocol [29]. COS-7 cells transiently expressing T7-tagged proteins (T7-TBC1D12, T7-TBC1D12-N, T7-TBC1D12-M, and T7-TBC1D12-TBC) were harvested and lysed in the lysis buffer, and total cell lysates were obtained by centrifugation at 17,400 *g* for 10 min at 4°C. A 10 μL wet volume each of glutathione-Sepharose 4B beads (GE Healthcare) coupled with GST fusion proteins was incubated with 100 μL of COS-7 cell lysates containing each T7-tagged protein. The beads were then washed 3 times with a washing buffer (50 mM HEPES-KOH, pH 7.2, 150 mM NaCl, 1 mM MgCl_2_, and 0.2% Triton X-100), and proteins trapped by the beads were subjected to SDS-PAGE followed by immunoblotting as described above.

### GAP assays

The assays for GAP activity of TBC1D12 and TBC1D25/OATL1 were performed as described previously with slight modifications [20]. In brief, a 200 pmol amount of purified GST-Rab11A (or GST-Rab33B) protein was incubated at 30°C for 10 min with 6.7 pmol of [α-^32^P]GTP (Perkin-Elmer Corp., Waltham, MA) and 800 pmol of cold GTP (Sigma-Aldrich) in 25 mM Tris-HCl, pH 7.5, 50 mM NaCl, 2.5 mM EDTA, and 0.5 mg/mL BSA. After adding MgCl_2_ (final concentration, 10 mM), the mixture was passed through a PD-10 column (GE Healthcare) filled with Sephadex G-25 (GE Healthcare), and the 3.0–3.5 mL eluted fractions were collected. The GTPase activity reaction was initiated by adding 5 pmol of purified GST-TBC protein (or GST alone as a control) to 10 μL aliquots of the fraction (equivalent of 2 pmol of Rab protein), and the reaction mixture was incubated at 30°C for 20 min. The reaction was halted by adding an equal volume of a stop buffer (20 mM EDTA and 0.4% SDS), and the mixture was incubated at 70°C for 5 min. A 1 μL sample was dropped on a thin-layer chromatography (TLC) plate (Merck Biosciences) and developed in 0.5 mM LiCl and 1 M formic acid. The amounts of GTP and GDP were determined with an FLA-3000 Bioimaging analyzer (FUJIFILM, Tokyo, Japan) and an imaging plate.

### Neurite outgrowth assays

Neurite outgrowth assays were performed as described previously [30]. In brief, 24 h after transfection PC12 cells were treated with 50 ng/mL of β-nerve growth factor (NGF; Merck Millipore) for 36 h. The cells were then fixed, and both fluorescence and bright-field images of enhanced green fluorescent protein (EGFP)-expressing cells were obtained at random with a confocal fluorescence microscope. The total neurite length of each transfected cell in bright-filed images was measured manually using the multi-line tool and Region Measurements dialogue in MetaMorph software (Molecular Devices, Sunnyvale, CA).

### Statistical analysis

The data were statistically analyzed by performing Student’s unpaired *t*-test, the Tukey-Kramer test, or the Dunnett test. The single asterisks (*), double asterisks (**), and triple asterisks (***) in the bar graphs indicate *p* values of <0.05, <0.01, and <0.001, respectively, and *p* values >0.05 are indicated by not significant (NS).

## Results

### Screening for TBC proteins that colocalize with transferrin receptor in MEFs

**Fig 1:**
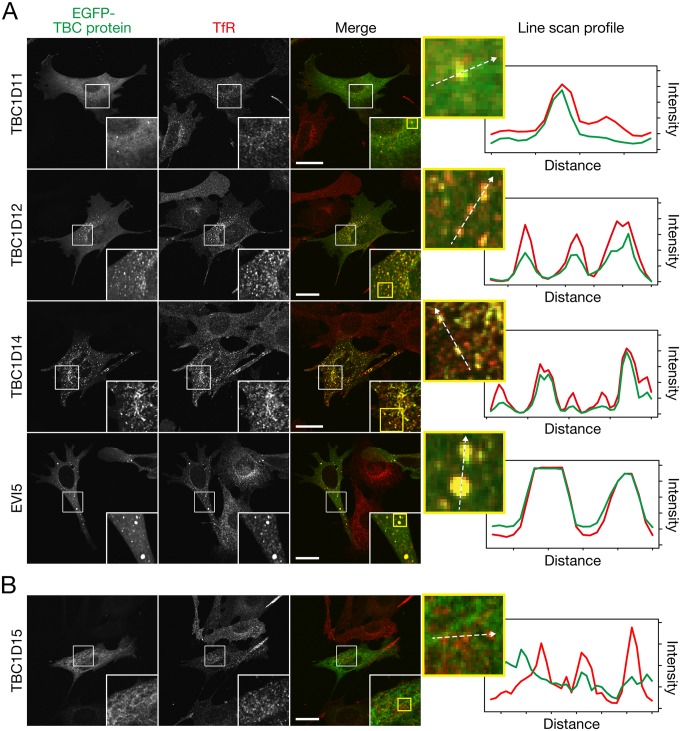
Identification of TBC proteins that specifically localize on recycling endosomes in MEFs. (A) Typical images of four TBC proteins that clearly colocalized with TfR (a recycling endosome marker). MEFs transiently expressing EGFP-TBC proteins were immunostained with anti-TfR antibody (1/250 dilution), and the stained cells were examined with a confocal fluorescence microscope. The insets show magnified views of the boxed areas. The line scan profiles (broken arrows in the far right column) were acquired by using ImageJ software. (B) A typical image of TBC protein showing no colocalization with TfR. Scale bars, 40 μm. The images of all other TBC proteins are summarized in S1 Fig.

To identify novel TBC proteins that regulate trafficking in recycling endosomes, we comprehensively screened all 43 mammalian TBC proteins that have been identified thus far for proteins that colocalize with TfR, a well-known recycling endosome marker, in MEFs. We expressed 43 different EGFP-tagged human or mouse TBC proteins in MEFs and investigated colocalization with endogenous TfR (S1 Fig). The results showed that four TBC proteins, i.e., TBC1D11, TBC1D12, TBC1D14, and EVI5, clearly colocalized with TfR on dot-like structures around the nucleus (Fig 1A), three TBC proteins, TBC1D2B, TBC1D23, and TBC1D25, partially colocalized with TfR (S1 Fig, arrows), and others, e.g., TBC1D15, did not (Fig 1B). Intriguingly, some large punctate TfR structures were often observed in MEFs expressing EVI5 (Fig 1A, bottom panels), indicating that EVI5 affects the recycling or trafficking of TfR. Since EVI5 has been shown to possess Rab11-GAP activity [31], it is highly possible that overexpressed EVI5 protein inactivates Rab11, thereby leading to inhibition of TfR recycling and its subsequent accumulation at large punctate structures. Because putative functions have already been reported for three of the four TBC proteins (TBC1D11, TBC1D14, and EVI5) [31–35], we focused our attention on the function of TBC1D12 on recycling endosomes.

### TBC1D12 localizes on Rab11-positive recycling endosomes in a Rab11-dependent manner

**Fig 2:**
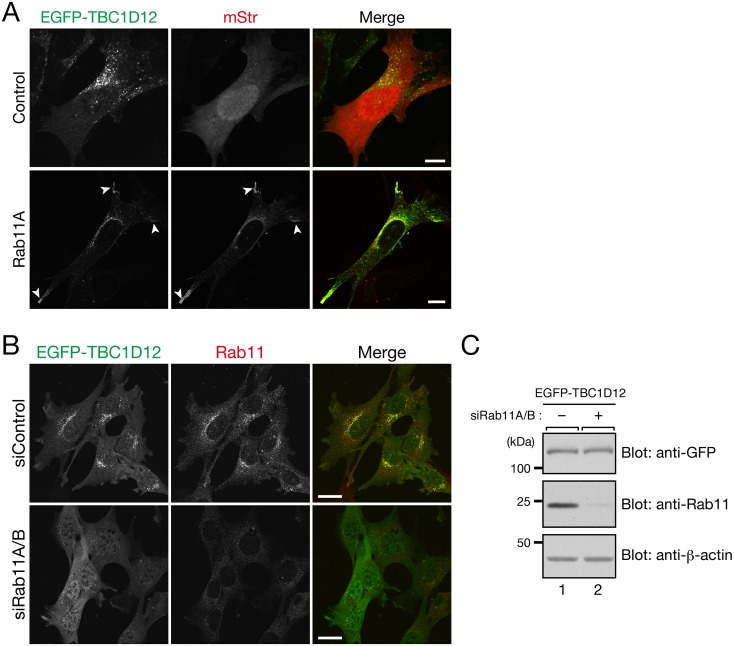
Rab11 recruits TBC1D12 to Rab11-positive recycling endosomes. (A) Overexpression of Rab11 altered TBC1D12 localization from around the nucleus to the cell protrusions (arrowheads). MEFs transiently expressing both EGFP-TBC1D12 and mStr-Rab11A (or mStr alone as a control) were examined with a confocal fluorescence microscope. Scale bars, 10 μm. (B) Knockdown of Rab11A/B caused dispersion of TBC1D12 into the cytosol. MEFs stably expressing T7-TBC1D12 were transfected with control siRNA (siControl) or Rab11A/B siRNAs (siRab11A/B) and immunostained with anti-TBC1D12 antibody (1/300 dilution) and anti-Rab11 antibody (1/200 dilution), and the stained cells were examined with a confocal fluorescence microscope. Scale bars, 20 μm. (C) Knockdown of Rab11A/B in MEFs as revealed by immunoblotting. The band intensity of Rab11 in siRab11A/B-treated cells was 6.7% of its band intensity in the control cells. Total cell lysates of siControl-treated cells (lane 1) and siRab11A/B-treated cells (lane 2) were analyzed by 8% SDS-PAGE and immunoblotting with anti-GFP antibody (top panel; 1/500 dilution), anti-Rab11 antibody (middle panel; 1/500 dilution), and anti-β-actin antibody (bottom panel; 1/15,000 dilution). The positions of the molecular mass markers (in kDa) are shown on the left.

To confirm the recycling endosomal localization of TBC1D12, we compared the localizations between EGFP-TBC1D12 and Rab11, another recycling endosome marker [12], and the results showed that TBC1D12 clearly colocalized with both endogenous Rab11 and mStr-tagged Rab11A (Fig 2A, bottom row, and Fig 2B, top row). Intriguingly, when mStr-Rab11A was coexpressed with EGFP-TBC1D12, TBC1D12 signals were observed at the cell protrusions (Fig 2A, arrowheads in the bottom row), whereas when control mStr alone was coexpressed, TBC1D12 signals were observed only around the nucleus (Fig 2A, top row). This Rab11-dependent difference in TBC1D12 localization led us to hypothesize that Rab11 recruits TBC1D12 to recycling endosomes. To test our hypothesis we knocked down endogenous Rab11A and Rab11B (Rab11A/B) in MEFs (Fig 2C) and investigated TBC1D12 localization in the Rab11A/B-knockdown (KD) cells. As predicted by our hypothesis, knockdown of Rab11A/B induced dispersion of TBC1D12 into the cytosol. The disappearance of TBC1D12 signals around the nucleus was unlikely to have been caused by a reduced level of protein expression, because the level of EGFP-TBC1D12 protein expression in the Rab11A/B-KD cells was unaltered (Fig 2C, top panel). These results allowed us to conclude that TBC1D12 is recruited to Rab11-positive recycling endosomes in a Rab11-dependent manner.

### TBC1D12 interacts with GTP-Rab11 through its middle region

**Fig 3:**
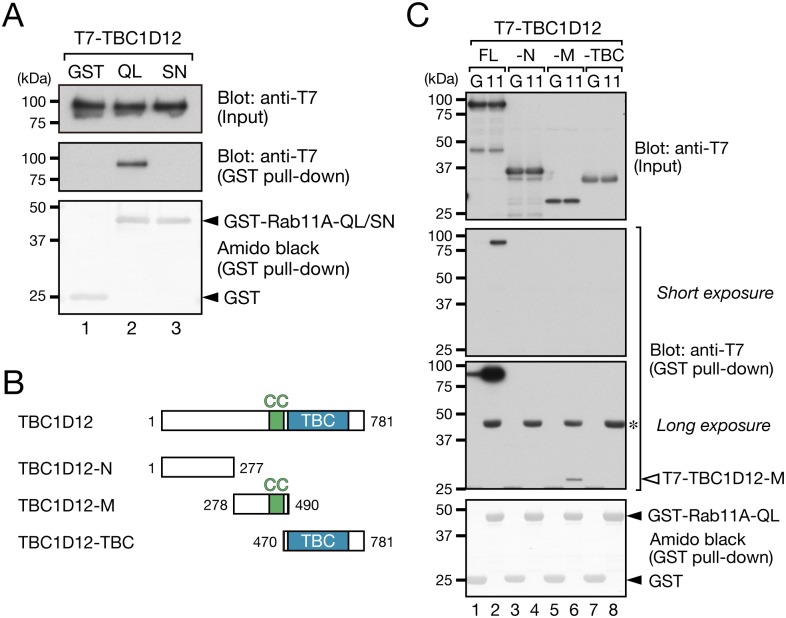
TBC1D12 interacts with Rab11 through its middle region. (A) TBC1D12 interacts with a constitutively active form that mimics the GTP-bound form of Rab11. Total lysates of COS-7 cells expressing T7-TBC1D12 were incubated with glutathione-Sepharose beads coupled with GST alone, GST-Rab11A-Q70L (indicated by QL), or GST-Rab11A-S25N (indicated by SN). A 2% volume of the reaction mixture (input) and proteins bound to the glutathione-Sepharose beads were analyzed by 10% SDS-PAGE and immunoblotting with HRP-conjugated anti-T7 tag antibody (top and middle panels), and the GST-fusion proteins were stained with amido black (bottom panel). The positions of the molecular mass markers (in kDa) are shown on the left. (B) Schematic representation of three deletion mutants of TBC1D12 (TBC1D12-N, -M, and -TBC). TBC domains and coiled-coil (CC) domains are shown as blue boxes and green boxes, respectively. (C) Total lysates of COS-7 cells expressing T7-TBC1D12 (indicated by FL, full-length), T7-TBC1D12-N, T7-TBC1D12-M, or T7-TBC1D12-TBC were incubated with glutathione-Sepharose beads coupled with GST alone (indicated by G) or GST-Rab11A-Q70L (indicated by 11). A 2% volume of the reaction mixture (input) and proteins bound to the beads were analyzed by 10% SDS-PAGE and immunoblotting with HRP-conjugated anti-T7 tag antibody (top three panels). The third panel corresponds to the longer exposure of the second panel. The GST-fusion proteins were stained with amido black (bottom panel). The asterisk indicates non-specific bands, and the positions of the molecular mass markers (in kDa) are shown on the left.

Next, we performed GST pull-down assays in an attempt to determine whether TBC1D12 interacts with Rab11. To do so, we purified GST-fusion proteins of a constitutively active form of Rab11A (GST-Rab11A-Q70L; mimics GTP-Rab11A) and its constitutively negative form (GST-Rab11A-S25N; mimics GDP-Rab11A) and coupled them to glutathione-Sepharose beads. The beads coupled to the GST-fusion proteins were then incubated with T7-tagged TBC1D12, and the bound TBC1D12 was detected by immunoblotting. As shown in Fig 3A (middle panel), TBC1D12 specifically interacted with Rab11A-Q70L and did not interact with Rab11A-S25N, indicating that TBC1D12 recognizes the GTP-bound form of Rab11 alone. Since, in principle, GAP recognizes the GTP-bound form of Rab as a substrate and some Rab-GAPs have been shown to physically interact with their substrate Rabs [20, 36], we assumed that a TBC/Rab-GAP domain of TBC1D12 interacts with Rab11A-Q70L. However, when we attempted to verify our assumption by producing three truncation mutants of TBC1D12, i.e., TBC1D12-N, TBC1D12-M, and TBC1D12-TBC (Fig 3B), and using GST-Rab11A-Q70L to perform GST pull-down assays as described above, we discovered that TBC1D12-M, which contains a coiled-coil domain that often serves as a Rab-binding domain [37], interacted with Rab11A-Q70L (Fig 3C, lane 6). By contrast, neither TBC1D12-N nor TBC1D12-TBC interacted with Rab11A-Q70L at all (Fig 3C, lanes 4 and 8). However, since the interaction between TBC1D12-M and Rab11A-Q70L is much weaker than the interaction between TBC1D12-FL and Rab11A-Q70L, an N-terminal or C-terminal flaking region of TBC1D12-M may be required for high-affinity Rab11 binding. These results indicated that TBC1D12 interacts with an active form of Rab11 mainly through its middle region, not through its TBC domain, suggesting that TBC1D12 is not a Rab11-GAP.

To determine whether TBC1D12 exhibits GAP activity toward Rab11, we measured the GAP activity of TBC1D12 toward Rab11A *in vitro* as described previously [20, 22]. As expected, TBC1D12 did not exhibit significant *in vitro* Rab11A-GAP activity, whereas the positive control, OATL1, exhibited strong Rab33B-GAP activity (S2 Fig). These results suggested that TBC1D12 acts as a Rab11-binding protein rather than as a Rab11-GAP.

### Overexpression of TBC1D12 has no effect on transferrin recycling or autophagy

Because TBC1D12 is recruited to recycling endosomes in a Rab11-binding-dependent manner (Figs 2 and 3), we attempted to determine whether TBC1D12 is involved in two well-known Rab11-dependent cellular events, i.e., transferrin (Tf) recycling and autophagy, both of which are observed in all cell types [5, 12]. Intriguingly, however, overexpression of TBC1D12 in MEFs affected neither Tf recycling as monitored by measuring the intensity of Alexa594-Tf staining (S3 Fig) nor starvation-induced autophagy as monitored by observing the formation of LC3 dots (S4 Fig).

### Overexpression of TBC1D12 in PC12 cells promotes neurite outgrowth independently of its GAP activity

**Fig 4:**
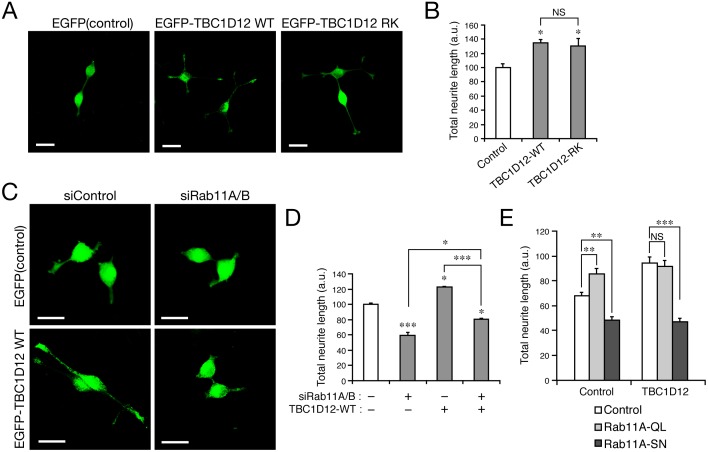
Overexpression of TBC1D12 in PC12 cells promotes neurite outgrowth independently of its GAP activity. (A) Overexpression of either TBC1D12 wild-type (WT) or its GAP-activity-deficient mutant (RK) promoted neurite outgrowth of PC12 cells. Typical images of PC12 cells expressing EGFP alone (control), EGFP-TBC1D12-WT, or EGFP-TBC1D12-RK. After NGF stimulation for 36 h the cells were fixed, immunostained with anti-GFP antibody (1/500 dilution), and examined with a fluorescence microscope. Scale bars, 30 μm. (B) The total neurite length of each cell in (A) after NGF stimulation for 36 h was measured with MetaMorph software (n >100). The total neurite length of each sample was normalized to that of the control cells. Error bars indicate the SEMs of the data from three independent experiments. No significant difference was observed between the WT and RK mutant. (C) TBC1D12 promotes neurite outgrowth in a Rab11-dependent manner. Typical images of PC12 cells expressing EGFP alone (control) or EGFP-TBC1D12-WT together with siControl or siRab11A/B. After NGF stimulation for 36 h the cells were fixed, immunostained with anti-GFP antibody (1/500 dilution), and examined with a fluorescence microscope. Scale bars, 30 μm. (D) The total neurite length of each cell in (C) after NGF stimulation for 36 h was measured with MetaMorph software (n >100). The total neurite length of each sample was normalized to that of the control cells. Error bars indicate the SEMs of the data from three independent experiments. *, *p* <0.05; **, *p* <0.01; ***, *p* <0.001; NS, not significant; and a.u., arbitrary unit. (E) Rab11-S25N (SN), but not Rab11-Q70L (QL), inhibits neurite outgrowth of both control cells and TBC1D12-expressing cells. Typical images are shown in S7 Fig. The total neurite length of each cell after NGF stimulation for 36 h was measured as described in (D). Error bars indicate the SEMs of the data from ≥79 cells. ***, *p* <0.001; **, *p* <0.01; NS, not significant. Although both positive and negative effects of Rab11-Q70L (or a positive effect of S25N) on neurite/axon outgrowth have been reported previously [10, 39], under our experimental conditions expression of Rab11-Q70L and Rab11-S25N in PC12 cells promoted and inhibited, respectively, NGF-stimulated neurite outgrowth.

Since TBC1D12 may not contribute to the well-known Rab11-dependent cellular events, i.e., transferrin recycling and autophagy, we turned our attention to the unique functions of Rab11 on recycling endosomes in a specialized cell type and focused on neurite outgrowth [6–11]. In the next set of experiments, we used PC12 cells as a model to investigate whether TBC1D12 participates in neurite outgrowth because PC12 cells endogenously express TBC1D12 (S5C Fig; anti-TBC1D12 antibody specifically recognized a single band around 80 kDa that almost corresponded to the calculated molecular mass of TBC1D12). When wild-type (WT) TBC1D12 was overexpressed in PC12 cells, TBC1D12-WT partially, but significantly colocalized with endogenous Rab11 in the perinuclear region (S5A Fig, arrows), and it significantly promoted NGF-stimulated neurite outgrowth of PC12 cells (Fig 4A and 4B). To rule out the possibility that the promotion of neurite outgrowth by overexpression of TBC1D12-WT was attributable to its GAP activity, we produced a GAP-activity-deficient TBC1D12 mutant, named TBC1D12-RK, in which the catalytic Arg residue in the TBC domain is replaced by Lys [38], and we overexpressed it in PC12 cells. As shown in Fig 4A and 4B, overexpression of TBC1D12-RK in PC12 cells promoted NGF-stimulated neurite outgrowth, the same as TBC1D12-WT did. No significant difference in neurite outgrowth was observed between the WT and RK mutant. Moreover, no difference in protein expression in PC12 cells was observed between the WT and RK mutant (S6 Fig). We therefore concluded that overexpression of TBC1D12 promotes neurite outgrowth of PC12 cells independently of its GAP-activity.

We also investigated whether the TBC1D12-overexpression-dependent neurite outgrowth was related to Rab11 by knocking down endogenous Rab11A/B or by co-expressing Rab11A mutants (Q70L and S25N) in TBC1D12-expessing PC12 cells and measuring the total length of their neurites. As expected, knockdown of Rab11A/B almost completely eliminated the promoting effect of TBC1D12 (Fig 4C and 4D). Similarly, co-expression of Rab11-S25N with TBC1D12 eliminated the promoting effect of TBC1D12, whereas co-expression of Rab11-Q70L and TBC1D12 was found not to have an additive effect on neurite outgrowth when compared to their solo expression (Fig 4E and S7 Fig). Expression of TBC1D12 (or Rab11-Q70L) alone may maximally activate the TBC1D12–Rab11 pathway to promote neurite outgrowth. Taken together, these results suggest that overexpression of TBC1D12 in PC12 cells promotes neurite outgrowth in a GTP-Rab11-dependent manner.

### Knockdown of endogenous TBC1D12 in PC12 cells promoted neurite outgrowth

**Fig 5:**
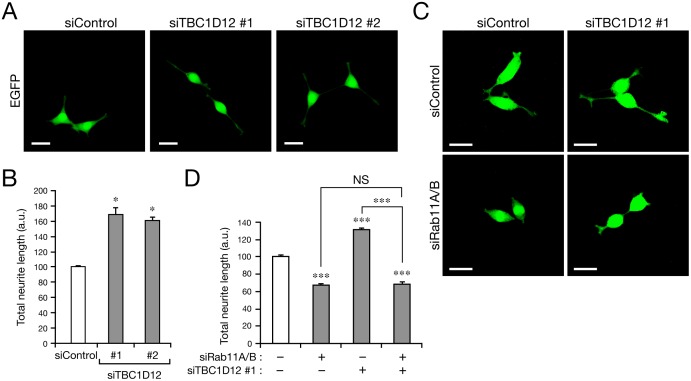
Knockdown of TBC1D12 in PC12 cells promotes neurite outgrowth. (A) Knockdown of TBC1D12 in PC12 cells promoted neurite outgrowth. Typical images of PC12 cells expressing EGFP together with either siControl, siTBC1D12 #1, or siTBC1D12 #2. After NGF stimulation for 36 h the cells were fixed and examined with a confocal fluorescence microscope. Scale bars, 30 μm. The knockdown efficiency of siTBC1D12 #1/2 is shown in S5C Fig. (B) The total neurite length of each cell in (A) after NGF simulation for 36 h was measured with MetaMorph software (n >100). The total neurite length of each sample was normalized to that of the control cells. Error bars indicate the SEMs of the data from three independent experiments. *, *p* <0.05; and a.u., arbitrary unit. (C) Promotion of neurite outgrowth of TBC1D12-KD cells occurred in a Rab11-dependent manner. Typical images of PC12 cells expressing EGFP together with either siControl, siTBC1D12 #1 + siControl, siTBC1D12 #1 + siRab11A/B, or siRab11A/B. After NGF stimulation for 36 h the cells were fixed and then examined with a fluorescence microscope. Scale bars, 30 μm. (D) The total neurite length of each cell in (C) after NGF stimulation for 36 h was measured with MetaMorph software (n >100). The total neurite length of each sample was normalized to that of the control cells. Error bars indicate the SEMs of the data from three independent experiments. ***, *p* <0.001; NS, not significant; and a.u., arbitrary unit.

Finally, we investigated whether TBC1D12 endogenously expressed in PC12 cells is actually involved in neurite outgrowth. To do so, we prepared two independent siRNAs against rat TBC1D12 and confirmed that the intensity of the TBC1D12 band in PC12 cells was dramatically reduced after treatment with the specific siRNAs (S5C Fig). By using these siRNAs, we knocked down endogenous TBC1D12 in PC12 cells and evaluated their effect on NGF-stimulated neurite outgrowth. We expected that knockdown and overexpression of TBC1D12 to have opposite effects, but TBC1D12 knockdown actually promoted neurite outgrowth of PC12 cells (Fig 5A and 5B), the same as overexpression did (Fig 4A and 4B). It should be noted, however, that the promotion of neurite outgrowth of the TBC1D12-KD cells occurred in a Rab11-dependent manner, i.e., knockdown of Rab11A/B in TBC1D12-KD cells almost completely cancelled out the promoting effect by TBC1D12 knockdown (Fig 5C and 5D). Although exactly why both overexpression and knockdown of TBC1D12 had the same effect is unknown (see Discussion below), our findings in this study indicated that TBC1D12 is a novel Rab11-dependent modulator of neurite outgrowth of PC12 cells.

## Discussion

In the present study, we for the first time comprehensively screened TBC proteins for proteins that specifically localize on recycling endosomes by using TfR as a marker, and we succeeded in identifying TBC1D12 as a novel recycling endosome-resident protein (Fig 1 and S1 Fig). Intriguingly, recruitment of TBC1D12 to recycling endosomes is entirely Rab11-dependent in MEFs (Fig 2) and TBC1D12 has the ability to bind Rab11 through its CC-domain-containing middle region, not through its TBC domain (Fig 3). Although TBC1D12 contains a putative Rab-GAP/TBC domain at its C terminus, Rab11 is unlikely to be its substrate, because TBC1D12 did not exhibit significant Rab11-GAP activity *in vitro* (S2 Fig). These findings suggested that TBC1D12 functions as a Rab11-binding protein rather than as a Rab11-GAP. Because some TBC proteins have been shown to function as effector molecules for small GTPases [5, 33, 40] and TBC1D12 interacts with the active, GTP-bound form of Rab11A (Fig 3A), it is tempting to speculate that TBC1D12 functions as a Rab11 effector. We noted that similar active Rab11 binding has also been reported in relation to TBC1D14, which is phylogenetically similar to TBC1D12 [18, 34]. Because of their high sequence similarity, TBC1D12 and TBC1D14 may recognize active Rab11 by a similar mechanism, but they clearly have different functions at least in autophagy, because TBC1D14 modulates autophagy [34] and TBC1D12 does not (S4 Fig). Further research is necessary to determine whether TBC1D12 acts as an actual Rab11 effector during neurite outgrowth.

One of the most unexpected findings in this study is that TBC1D12 is not involved in either of the two well-known Rab11-dependent cellular events, Tf recycling (S3 Fig) and starvation-induced autophagy (S4 Fig), both of which have been observed in all cell types. Instead, TBC1D12 was found to be involved in a Rab11-dependent cellular event in a specialized cell type, i.e., in neurite outgrowth of PC12 cells (Figs 4 and 5). A number of studies have shown that Rabs and their effectors on recycling endosomes regulate neurite outgrowth (reviewed in [41]), but to our knowledge TBC1D12 is the first endosomal TBC protein that has been demonstrated to modulate neurite outgrowth. We speculate that TBC1D12 is involved in membrane trafficking from recycling endosomes in the cell body to the tips of neurites (i.e., supplies proteins and lipids to neurite tips) by modulating the function of Rab11.

The results of our study gave rise to several questions about the function of TBC1D12 that need to be answered in our next study. The first question is: Why does TBC1D12 modulate neurite outgrowth of PC12 cells but not modulate Tf recycling or autophagy? We speculate that TBC1D12 recruits an important neurite outgrowth factor to Rab11-positive recycling endosomes, and that factor may be expressed only in neuronal cells and not contribute to Tf recycling or autophagy in MEFs. The second question is: Why do both overexpression and knockdown of TBC1D12 in PC12 cells result in the same phenotype, i.e., promotion of neurite outgrowth? We do not know exactly why, but similar observations have been made in regard to Rab32/38 effector Varp and Rab9 effector RUTBC1, i.e., both their knockdown and overexpression have been found to result in the same defect in melanogenic enzyme transport in melanocytes [42, 43]. One possible answer to this question is that in the absence of TBC1D12 Rab11 interacts with other Rab11 effectors [11, 39, 44] that promote neurite outgrowth more efficiently than TBC1D12 does. The final question is: Does TBC1D12 actually function as a Rab-GAP despite the fact that both the wild-type and RK mutant of TBC1D12 promoted neurite outgrowth of PC12 cells (Fig 4A and 4B). The actual target of TBC1D12 is unknown, but its target would have to be a Rab that is also recruited to recycling endosomes and functions before Rab11. We speculate that the candidate Rab and Rab11 function sequentially in the same membrane trafficking pathway and constitute a Rab effector–Rab-GAP cascade [45, 46]. Active Rab11 recruits TBC1D12 as a Rab11-binding protein to recycling endosomes, where the candidate Rab is also present and functions before Rab11, and TBC1D12 then functions as a GAP by inactivating the candidate Rab. It would be interesting to investigate whether the GAP activity of TBC1D12 is involved in other Rab11-dependent cellular events, e.g., in cytokinesis and cell migration, which we did not investigate in this study.

In conclusion, we identified TBC1D12 as a novel Rab11-binding protein on recycling endosomes. Despite the fact that TBC1D12 has Rab11 binding ability, TBC1D12 expression had no effect on Tf recycling or autophagy in MEF cells and specifically promoted neurite outgrowth of PC12 cells. Our results highlight the GAP-activity-independent but Rab11-dependent role of TBC1D12 in neurite outgrowth.

## Supporting information

S1 Fig Summary of the results of screening for TBC proteins that localize on TfR-positive recycling endosomes in MEFs. (A) Typical images of MEFs transiently expressing EGFP alone (control) or TBC proteins. The cells were immunostained with anti-TfR antibody (1/250 dilution) and examined with a confocal fluorescence microscope. The arrows indicate partial colocalization between TBC proteins and TfR. The insets show magnified views of the boxed areas. Scale bars, 40 μm. (B) Summary of colocalization between TBC proteins and TfR. The degrees of colocalization between TBC proteins and TfR are shown in the second column. -, no colocalization; *, partial colocalization; ***, very high colocalization; and ND, not determined because of a low level of TBC protein expression in MEFs. At least 30 cells from three independent experiments were examined to evaluate colocalization between TBC proteins and TfR.
(PDF)

S2 Fig TBC1D12 does not exhibit Rab11A-GAP activity *in vitro*. (A) No Rab11A-GAP activity of TBC1D12. Purified T7-GST-TBC1D12 (or T7-GST as a control) was incubated for 20 min with purified Rab11A that had been loaded with [α-^32^P]GTP. (B) Rab33B-GAP activity of OATL1. Purified T7-GST-OATL1 (or T7-GST as a control) was incubated for 20 min with purified Rab33B that had been loaded with [α-^32^P]GTP. OATL1 exhibited significant *in vitro* Rab33B-GAP activity. Error bars indicate the SEMs of data from three experiments. **, *p* <0.01; and NS, not significant.
(PDF)

S3 Fig Effect of TBC1D12 overexpression on Tf recycling in MEF cells. (A) Overexpression of TBC1D12 did not affect Alexa594-Tf recycling. MEFs transiently expressing EGFP alone (control), EGFP-Rab11-S25N, or EGFP-TBC1D12 were incubated in DMEM containing 5 μg/mL Alexa594-Tf for 1 h on ice, and after incubation for 0 min or 30 min in DMEM containing 10% FBS, the cells were fixed and examined with a confocal fluorescence microscope. Scale bars, 20 μm. (B) The intensity of Alexa594-Tf staining in individual cells was measured with the ImageJ software (n >40). Error bars indicate the SEMs of data from >40 cells. ***, *p* <0.001; and NS, not significant.
(PDF)

S4 Fig Effect of TBC1D12 overexpression on starvation-induced autophagy in MEF cells. (A) Overexpression of TBC1D12 did not affect LC3 dot numbers under starved conditions. MEFs transiently expressing EGFP alone (control) or EGFP-TBC1D12 were fixed after incubation for 2 h in EBSS. The cells were immunostained with anti-LC3 antibody (1/250 dilution) and examined with a confocal fluorescence microscope. Scale bars, 40 μm. (B) The mean numbers of LC3-positive dots per cell in (A) are shown. Error bars indicate the SEMs of data from n = 24 (control) and n = 19 (EGFP-TBC1D12). NS, not significant.
(PDF)

S5 Fig Expression and localization of TBC1D12 in PC12 cells. (A) TBC1D12 partially colocalized with endogenous Rab11 in the perinuclear region of PC12 cells. Typical images of PC12 cells expressing EGFP alone or EGFP-TBC1D12. PC12 cells transiently expressing EGFP alone (control; top row) or EGFP-TBC1D12 (bottom row) were immunostained with anti-Rab11 antibody (1/200 dilution), and the stained cells were examined with a confocal fluorescence microscope. The inset shows magnified views of the boxed area. The arrows indicate the sites of colocalization between EGFP-TBC1D12 and Rab11. Their colocalization is less evident in PC12 cells than in the MEFs shown in Fig 1A, presumably because the Rab11 in PC12 cells also interacts with other effectors [11, 39, 44] or because the TBC1D12–Rab11 interaction in PC12 cells is unstable and occurs transiently. Alternatively, the amount of GTP-Rab11 in PC12 cells may be much lower than in MEFs. Scale bars, 5 μm. (B) The Pearson’s correlation coefficient value for colocalization between TBC1D12 and Rab11 in each cell was measured with ImageJ software. Error bars indicate the SEMs of the data from ≥10 cells. ***, *p* <0.001. (C) Endogenous expression of TBC1D12 in PC12 cells and its knockdown by specific siRNAs as revealed by immunoblotting. The band intensity of TBC1D12 in siTBC1D12 #1-treated cells and siTBC1D12 #2-treated cells was 31.0% (lane 2) and 22.4% (lane3), respectively, of its band intensity in the control cells. Total lysates of PC12 cells transfected with siControl, siTBC1D12 #1, or siTBC1D12 #2 were analyzed by 10% SDS-PAGE and immunoblotting with anti-TBC1D12 antibody (top panel; 1/500 dilution) and anti-β-actin antibody (bottom panel; 1/20,000 dilution). The positions of the molecular mass markers (in kDa) are shown on the left.
(PDF)

S6 Fig Expression of the recombinant TBC1D12-WT and TBC1D12-RK in PC12 cells. Total lysates of PC12 cells transiently expressing EGFP-TBC1D12-WT or EGFP-TBC1D12-RK were analyzed by 10% SDS-PAGE and immunoblotting with HRP-conjugated anti-GFP antibody (top panel; 1/5000 dilution) and anti-β-actin antibody (bottom panel; 1/20,000 dilution). The positions of the molecular mass markers (in kDa) are shown on the left.
(PDF)

S7 Fig Typical images of PC12 cells expressing either EGFP alone (control) or EGFP-TBC1D12-WT in the presence of mStr alone (control), mStr-Rab11-Q70L (QL) or mStr-Rab11-S25N (SN). After NGF stimulation for 36 h the cells were fixed and examined with a fluorescence microscope to determine the length of their neurites (see Fig 4E). Scale bars, 30 μm.
(PDF)
