# Supplementary material for: TBC1D12 is a novel Rab11-binding protein that modulates neurite outgrowth of PC12 cells
Source: PLoS One. 2017 Apr 6;12(4):e0174883. doi: 10.1371/journal.pone.0174883 (PMC5383037; doi:10.1371/journal.pone.0174883)
Supplement: S1 Fig — (A) Typical images of MEFs transiently expressing EGFP alone (control) or TBC proteins. The cells were immunostained with anti-TfR antibody (1/250 dilution) and examined with a confocal fluorescence microscope. The arrows indicate partial colocalization between TBC proteins and TfR. The insets show magnified views of the boxed areas. Scale bars, 40 μm. (B) Summary of colocalization between TBC proteins and TfR. The degrees of colocalization between TBC proteins and TfR are shown in the second column. -, no colocalization; *, partial colocalization; ***, very high colocalization; and ND, not determined because of a low level of TBC protein expression in MEFs. At least 30 cells from three independent experiments were examined to evaluate colocalization between TBC proteins and TfR. (PDF) [file pone.0174883.s001.pdf]

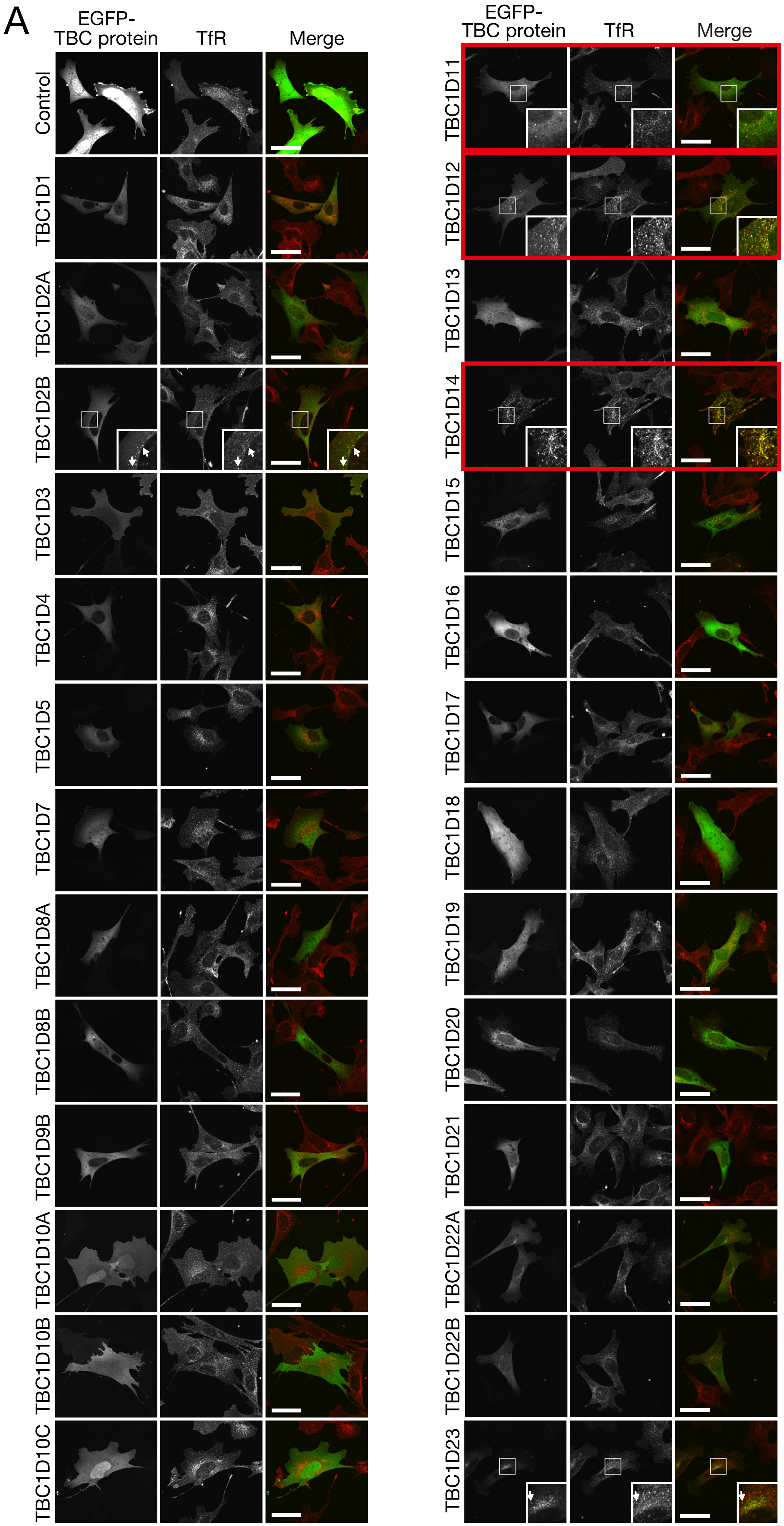
A
EGFP-
TBC protein
TfR
Merge
Control
TBC1D1
TBC1D2A
TBC1D2B
TBC1D3
TBC1D4
TBC1D5
TBC1D7
TBC1D8A
TBC1D8B
TBC1D9B
TBC1D10A
TBC1D10B
TBC1D10C
TBC1D11
TBC1D12
TBC1D13
TBC1D14
TBC1D15
TBC1D16
TBC1D17
TBC1D18
TBC1D19
TBC1D20
TBC1D21
TBC1D22A
TBC1D22B
TBC1D23

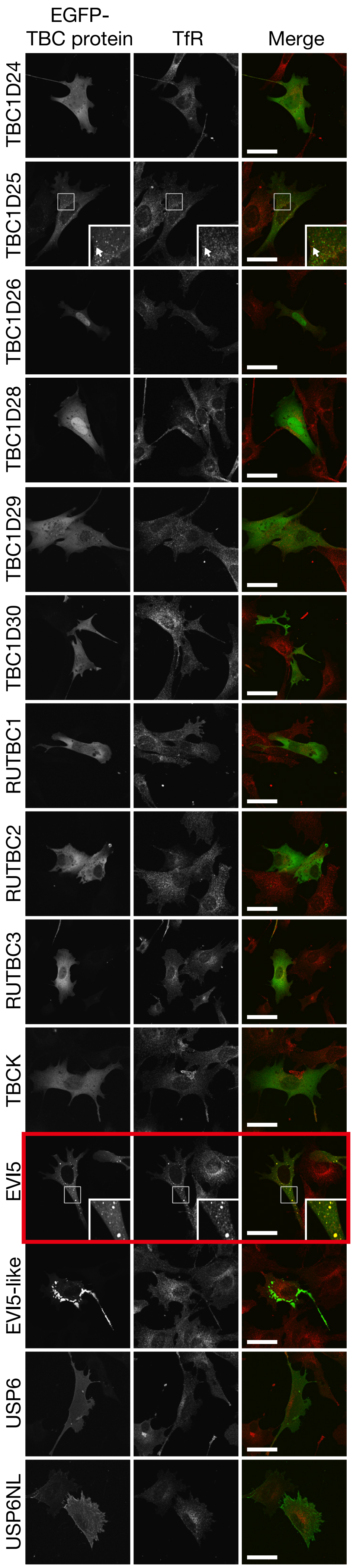

B

Gene symbol	Colocalization with TfR
TBC1D1	–
TBC1D2A	–
TBC1D2B	*
TBC1D3	–
TBC1D4	–
TBC1D5	–
TBC1D6	ND
TBC1D7	–
TBC1D8A	–
TBC1D8B	–
TBC1D9A	ND
TBC1D9B	–
TBC1D10A	–
TBC1D10B	–
TBC1D10C	–
TBC1D11	***
TBC1D12	***
TBC1D13	–
TBC1D14	***
TBC1D15	–
TBC1D16	–
TBC1D17	–
TBC1D18	–
TBC1D19	–
TBC1D20	–
TBC1D21	–
TBC1D22A	–
TBC1D22B	–
TBC1D23	*
TBC1D24	–
TBC1D25	*
TBC1D26	–
TBC1D28	–
TBC1D29	–
TBC1D30	–
RUTBC1	–
RUTBC2	–
RUTBC3	–
TBCK	–
EVI5	***
EVI5-like	–
USP6	–
USP6NL	–

S1 Fig. Summary of the results of screening for TBC proteins that localize on TfR-positive recycling endosomes in MEFs. (A) Typical images of MEFs transiently expressing EGFP alone (control) or TBC proteins. The cells were immunostained with anti-TfR antibody (1/250 dilution) and examined with a confocal fluorescence microscope. The arrows indicate partial colocalization between TBC proteins and TfR. The insets show magnified views of the boxed areas. Scale bars, 40 µm. (B) Summary of colocalization between TBC proteins and TfR. The degrees of colocalization between TBC proteins and TfR are shown in the second column. -, no colocalization; *, partial colocalization; ***, very high colocalization; and ND, not determined because of a low level of TBC protein expression in MEFs. At least 30 cells from three independent experiments were examined to evaluate colocalization between TBC proteins and TfR.
