# Supplementary material for: TBC1D12 is a novel Rab11-binding protein that modulates neurite outgrowth of PC12 cells
Source: PLoS One. 2017 Apr 6;12(4):e0174883. doi: 10.1371/journal.pone.0174883 (PMC5383037; doi:10.1371/journal.pone.0174883)
Supplement: S2 Fig — (A) No Rab11A-GAP activity of TBC1D12. Purified T7-GST-TBC1D12 (or T7-GST as a control) was incubated for 20 min with purified Rab11A that had been loaded with [α-32P]GTP. (B) Rab33B-GAP activity of OATL1. Purified T7-GST-OATL1 (or T7-GST as a control) was incubated for 20 min with purified Rab33B that had been loaded with [α-32P]GTP. OATL1 exhibited significant in vitro Rab33B-GAP activity. Error bars indicate the SEMs of data from three experiments. **, p <0.01; and NS, not significant. (PDF) [file pone.0174883.s002.pdf]

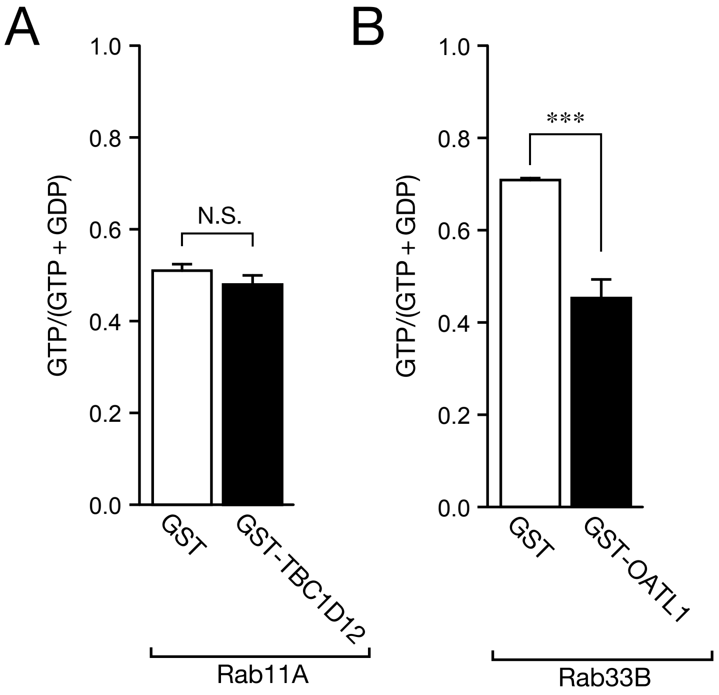

S2 Fig. TBC1D12 does not exhibit Rab11A-GAP activity *in vitro*. (A) No Rab11A-GAP activity of TBC1D12. Purified T7-GST-TBC1D12 (or T7-GST as a control) was incubated for 20 min with purified Rab11A that had been loaded with [α-^{32}P]GTP. (B) Rab33B-GAP activity of OATL1. Purified T7-GST-OATL1 (or T7-GST as a control) was incubated for 20 min with purified Rab33B that had been loaded with [α-^{32}P]GTP. OATL1 exhibited significant *in vitro* Rab33B-GAP activity. Error bars indicate the SEMs of data from three experiments. **, p <0.01; and NS, not significant.
