# Supplementary material for: TBC1D12 is a novel Rab11-binding protein that modulates neurite outgrowth of PC12 cells
Source: PLoS One. 2017 Apr 6;12(4):e0174883. doi: 10.1371/journal.pone.0174883 (PMC5383037; doi:10.1371/journal.pone.0174883)
Supplement: S3 Fig — (A) Overexpression of TBC1D12 did not affect Alexa594-Tf recycling. MEFs transiently expressing EGFP alone (control), EGFP-Rab11-S25N, or EGFP-TBC1D12 were incubated in DMEM containing 5 μg/mL Alexa594-Tf for 1 h on ice, and after incubation for 0 min or 30 min in DMEM containing 10% FBS, the cells were fixed and examined with a confocal fluorescence microscope. Scale bars, 20 μm. (B) The intensity of Alexa594-Tf staining in individual cells was measured with the ImageJ software (n >40). Error bars indicate the SEMs of data from >40 cells. ***, p <0.001; and NS, not significant. (PDF) [file pone.0174883.s003.pdf]

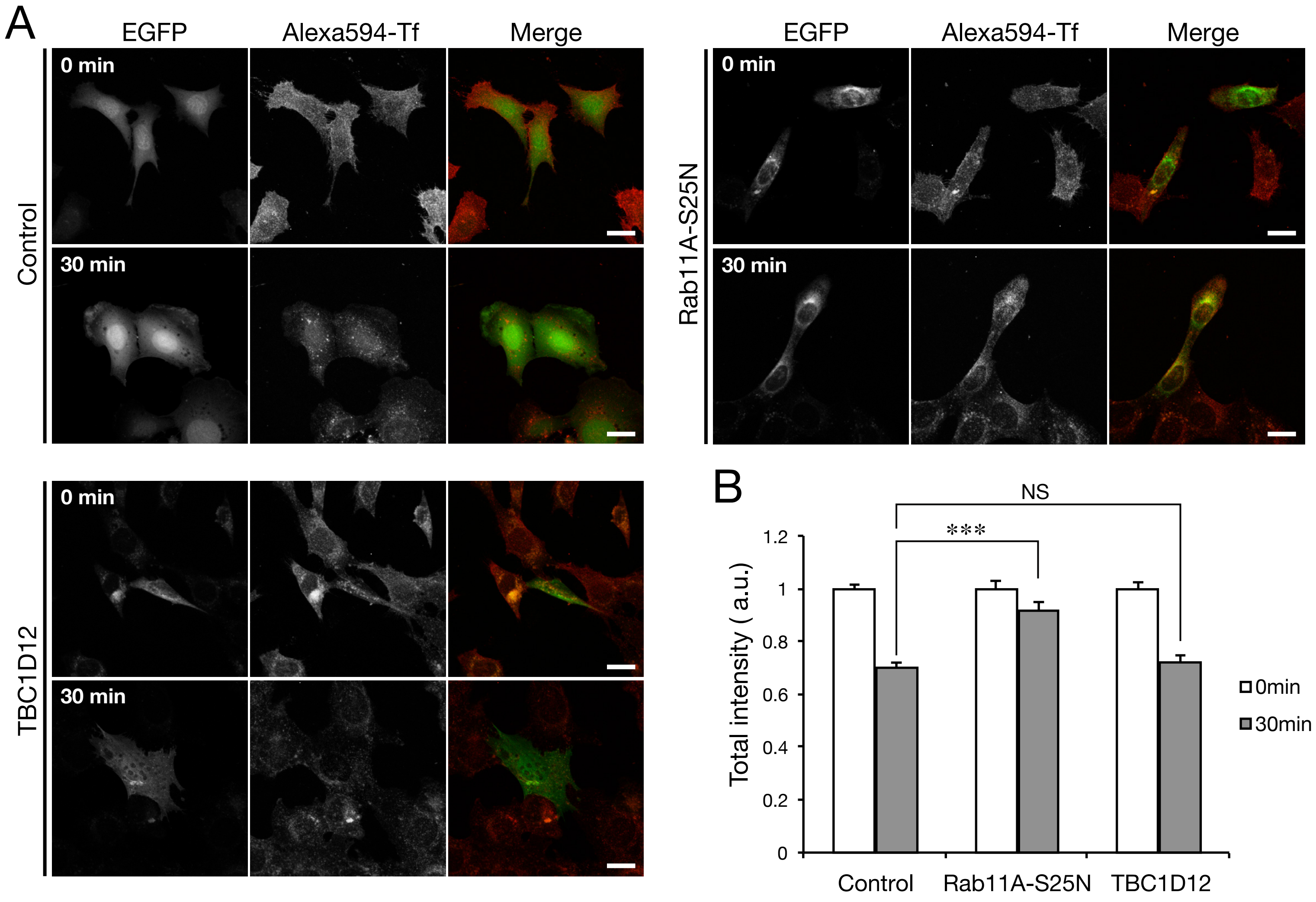

S3 Fig. Effect of TBC1D12 overexpression on Tf recycling in MEF cells. (A) Overexpression of TBC1D12 did not affect Alexa594-Tf recycling. MEFs transiently expressing EGFP alone (control), EGFP-Rab11-S25N, or EGFP-TBC1D12 were incubated in DMEM containing 5 μg/mL Alexa594-Tf for 1 h on ice, and after incubation for 0 min or 30 min in DMEM containing 10% FBS, the cells were fixed and examined with a confocal fluorescence microscope. Scale bars, 20 μm. (B) The intensity of Alexa594-Tf staining in individual cells was measured with the ImageJ software (n >40). Error bars indicate the SEMs of data from >40 cells. ***, *p* <0.001; and NS, not significant.
