# Supplementary material for: TBC1D12 is a novel Rab11-binding protein that modulates neurite outgrowth of PC12 cells
Source: PLoS One. 2017 Apr 6;12(4):e0174883. doi: 10.1371/journal.pone.0174883 (PMC5383037; doi:10.1371/journal.pone.0174883)
Supplement: S4 Fig — (A) Overexpression of TBC1D12 did not affect LC3 dot numbers under starved conditions. MEFs transiently expressing EGFP alone (control) or EGFP-TBC1D12 were fixed after incubation for 2 h in EBSS. The cells were immunostained with anti-LC3 antibody (1/250 dilution) and examined with a confocal fluorescence microscope. Scale bars, 40 μm. (B) The mean numbers of LC3-positive dots per cell in (A) are shown. Error bars indicate the SEMs of data from n = 24 (control) and n = 19 (EGFP-TBC1D12). NS, not significant. (PDF) [file pone.0174883.s004.pdf]

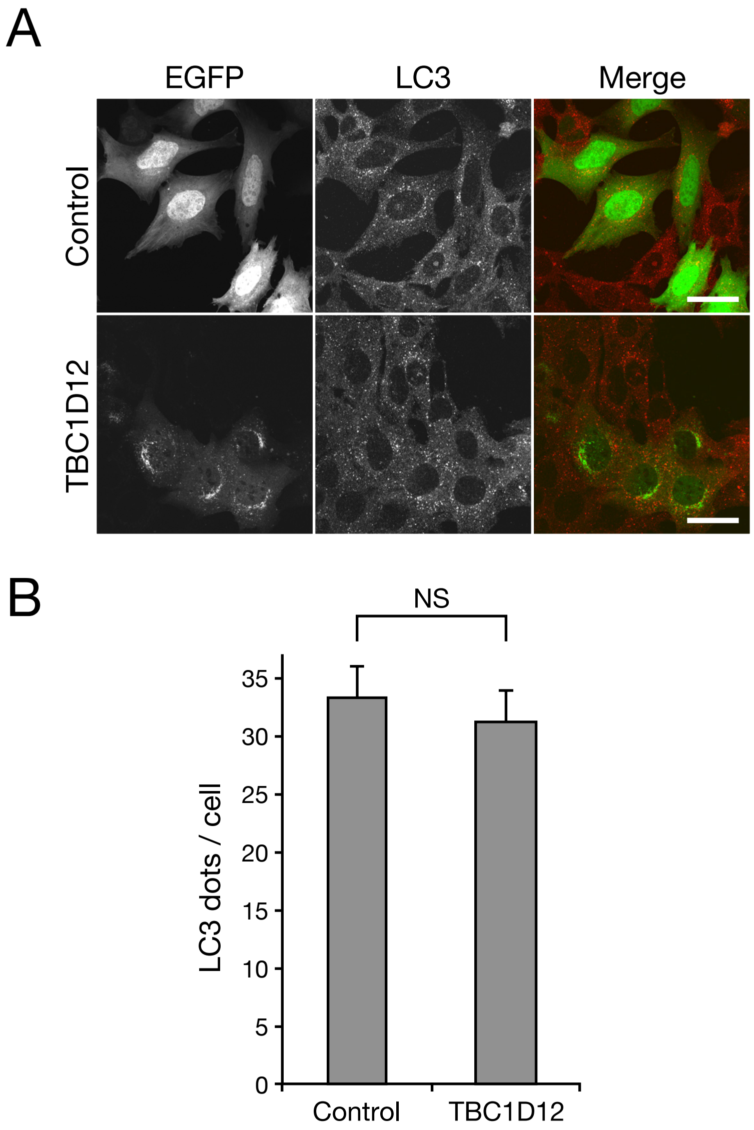

S4 Fig. Effect of TBC1D12 overexpression on starvation-induced autophagy in MEF cells. (A) Overexpression of TBC1D12 did not affect LC3 dot numbers under starved conditions. MEFs transiently expressing EGFP alone (control) or EGFP-TBC1D12 were fixed after incubation for 2 h in EBSS. The cells were immunostained with anti-LC3 antibody (1/250 dilution) and examined with a confocal fluorescence microscope. Scale bars, 40 μm. (B) The mean numbers of LC3-positive dots per cell in (A) are shown. Error bars indicate the SEMs of data from n = 24 (control) and n = 19 (EGFP-TBC1D12). NS, not significant.
