# Supplementary material for: TBC1D12 is a novel Rab11-binding protein that modulates neurite outgrowth of PC12 cells
Source: PLoS One. 2017 Apr 6;12(4):e0174883. doi: 10.1371/journal.pone.0174883 (PMC5383037; doi:10.1371/journal.pone.0174883)
Supplement: S5 Fig — (A) TBC1D12 partially colocalized with endogenous Rab11 in the perinuclear region of PC12 cells. Typical images of PC12 cells expressing EGFP alone or EGFP-TBC1D12. PC12 cells transiently expressing EGFP alone (control; top row) or EGFP-TBC1D12 (bottom row) were immunostained with anti-Rab11 antibody (1/200 dilution), and the stained cells were examined with a confocal fluorescence microscope. The inset shows magnified views of the boxed area. The arrows indicate the sites of colocalization between EGFP-TBC1D12 and Rab11. Their colocalization is less evident in PC12 cells than in the MEFs shown in Fig 1A, presumably because the Rab11 in PC12 cells also interacts with other effectors [11, 39, 44] or because the TBC1D12–Rab11 interaction in PC12 cells is unstable and occurs transiently. Alternatively, the amount of GTP-Rab11 in PC12 cells may be much lower than in MEFs. Scale bars, 5 μm. (B) The Pearson’s correlation coefficient value for colocalization between TBC1D12 and Rab11 in each cell was measured with ImageJ software. Error bars indicate the SEMs of the data from ≥10 cells. ***, p <0.001. (C) Endogenous expression of TBC1D12 in PC12 cells and its knockdown by specific siRNAs as revealed by immunoblotting. The band intensity of TBC1D12 in siTBC1D12 #1-treated cells and siTBC1D12 #2-treated cells was 31.0% (lane 2) and 22.4% (lane3), respectively, of its band intensity in the control cells. Total lysates of PC12 cells transfected with siControl, siTBC1D12 #1, or siTBC1D12 #2 were analyzed by 10% SDS-PAGE and immunoblotting with anti-TBC1D12 antibody (top panel; 1/500 dilution) and anti-β-actin antibody (bottom panel; 1/20,000 dilution). The positions of the molecular mass markers (in kDa) are shown on the left. (PDF) [file pone.0174883.s005.pdf]

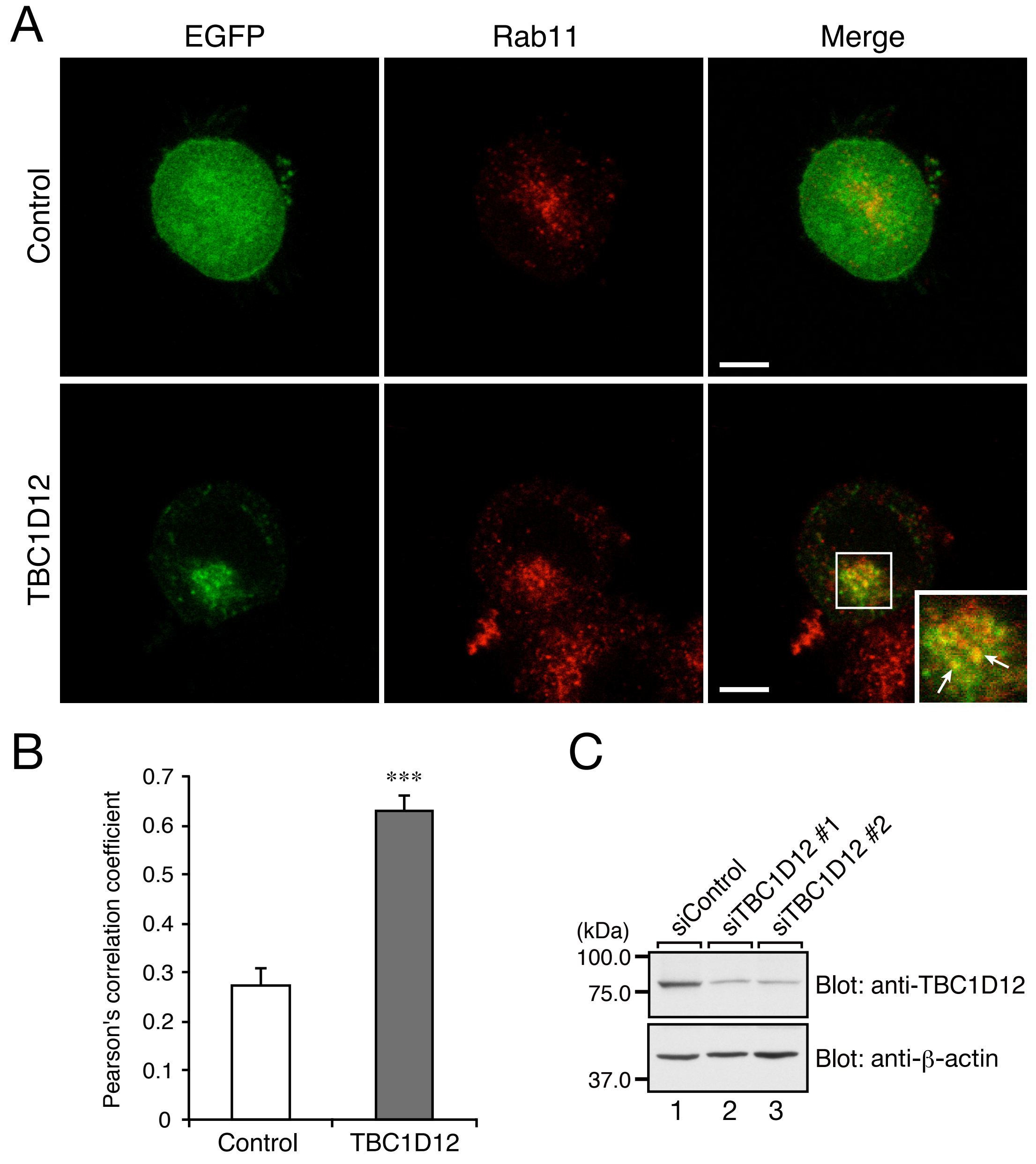

S5 Fig. Expression and localization of TBC1D12 in PC12 cells. (A) TBC1D12 partially colocalized with endogenous Rab11 in the perinuclear region of PC12 cells. Typical images of PC12 cells expressing EGFP alone or EGFP-TBC1D12. PC12 cells transiently expressing EGFP alone (control; top row) or EGFP-TBC1D12 (bottom row) were immunostained with anti-Rab11 antibody (1/200 dilution), and the stained cells were examined with a confocal fluorescence microscope. The inset shows magnified views of the boxed area. The arrows indicate the sites of colocalization between EGFP-TBC1D12 and Rab11. Their colocalization is less evident in PC12 cells than in the MEFs shown in Fig 1A, presumably because the Rab11 in PC12 cells also interacts with other effectors [11, 39, 44] or because the TBC1D12–Rab11 interaction in PC12 cells is unstable and occurs transiently. Alternatively, the amount of GTP-Rab11 in PC12 cells may be much lower than in MEFs. Scale bars, 5 μm. (B) The Pearson's correlation coefficient value for colocalization between TBC1D12 and Rab11 in each cell was measured with ImageJ software.

Error bars indicate the SEMs of the data from ≥10 cells. ***, *p* <0.001. (C) Endogenous expression of TBC1D12 in PC12 cells and its knockdown by specific siRNAs as revealed by immunoblotting. The band intensity of TBC1D12 in siTBC1D12 #1-treated cells and siTBC1D12 #2-treated cells was 31.0% (lane 2) and 22.4% (lane3), respectively, of its band intensity in the control cells. Total lysates of PC12 cells transfected with siControl, siTBC1D12 #1, or siTBC1D12 #2 were analyzed by 10% SDS-PAGE and immunoblotting with anti-TBC1D12 antibody (top panel; 1/500 dilution) and anti-β-actin antibody (bottom panel; 1/20,000 dilution). The positions of the molecular mass markers (in kDa) are shown on the left.
