# Supplementary material for: TBC1D12 is a novel Rab11-binding protein that modulates neurite outgrowth of PC12 cells
Source: PLoS One. 2017 Apr 6;12(4):e0174883. doi: 10.1371/journal.pone.0174883 (PMC5383037; doi:10.1371/journal.pone.0174883)
Supplement: S6 Fig — Total lysates of PC12 cells transiently expressing EGFP-TBC1D12-WT or EGFP-TBC1D12-RK were analyzed by 10% SDS-PAGE and immunoblotting with HRP-conjugated anti-GFP antibody (top panel; 1/5000 dilution) and anti-β-actin antibody (bottom panel; 1/20,000 dilution). The positions of the molecular mass markers (in kDa) are shown on the left. (PDF) [file pone.0174883.s006.pdf]

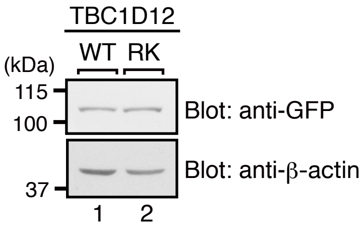

S6 Fig. Expression of the recombinant TBC1D12-WT and TBC1D12-RK in PC12 cells. Total lysates of PC12 cells transiently expressing EGFP-TBC1D12-WT or EGFP-TBC1D12-RK were analyzed by 10% SDS-PAGE and immunoblotting with HRP-conjugated anti-GFP antibody (top panel; 1/5000 dilution) and anti-β-actin antibody (bottom panel; 1/20,000 dilution). The positions of the molecular mass markers (in kDa) are shown on the left.
