# Supplementary material for: TBC1D12 is a novel Rab11-binding protein that modulates neurite outgrowth of PC12 cells
Source: PLoS One. 2017 Apr 6;12(4):e0174883. doi: 10.1371/journal.pone.0174883 (PMC5383037; doi:10.1371/journal.pone.0174883)
Supplement: S7 Fig — After NGF stimulation for 36 h the cells were fixed and examined with a fluorescence microscope to determine the length of their neurites (see Fig 4E). Scale bars, 30 μm. (PDF) [file pone.0174883.s007.pdf]

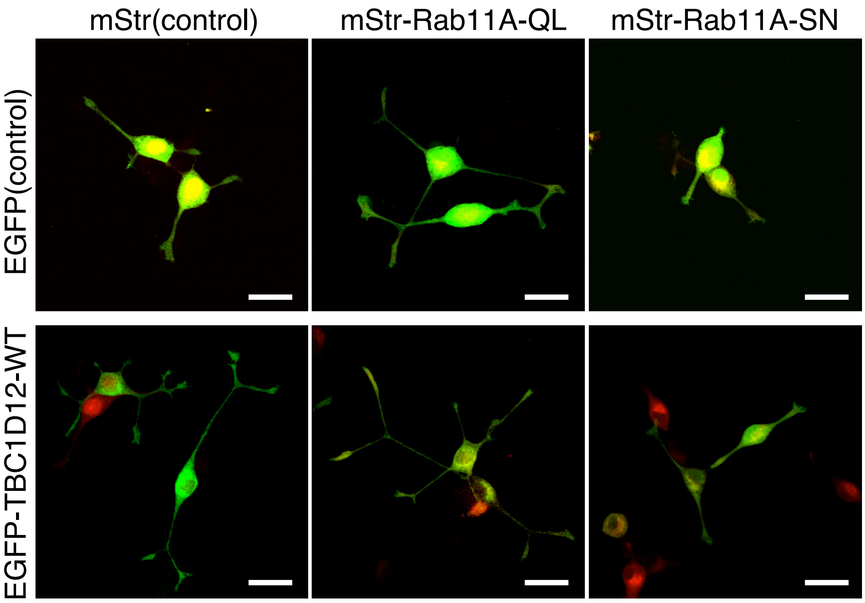

S7 Fig. Typical images of PC12 cells expressing either EGFP alone (control) or EGFP-TBC1D12-WT in the presence of mStr alone (control), mStr-Rab11-Q70L (QL) or mStr-Rab11-S25N (SN). After NGF stimulation for 36 h the cells were fixed and examined with a fluorescence microscope to determine the length of their neurites (see Fig. 4E). Scale bars, 30 μm.
